# Clinical outcomes associated with kidney function changes in anticoagulated atrial fibrillation patients: An ancillary analysis from the BOREALIS trial

**DOI:** 10.1002/joa3.12306

**Published:** 2020-02-06

**Authors:** Ying Bai, Alena Shantsila, Gregory Y. H. Lip

**Affiliations:** ^1^ Liverpool Centre for Cardiovascular Science University of Liverpool and Liverpool Heart & Chest Hospital Liverpool United Kingdom; ^2^ Cardiovascular Center Beijing Tongren Hospital Capital Medical University Beijing China; ^3^ Aalborg Thrombosis Research Unit Department of Clinical Medicine Aalborg University Aalborg Denmark

**Keywords:** deteriorated CKD‐EPI eGFR change, major bleeding, all‐cause death, preserved CKD‐EPI eGFR change, stroke/systemic embolism

## Abstract

**Background:**

Patients with atrial fibrillation (AF) and chronic kidney disease represent a high‐risk group for thromboembolism and bleeding.

**Aims:**

To explore the relationship between kidney function changes and outcomes of stroke/systemic embolism (SE), major bleeding and all‐cause death in anticoagulated AF patients participating in the BOREALIS trial comparing efficacy and safety of once‐weekly s.c. idrabiotaparinux to that of warfarin.

**Methods:**

Changes in kidney function by estimated glomerular filtration rate (eGFR) were calculated using the Chronic Kidney Disease Epidemiology Collaboration equation in 2765 AF patients. Trial adjudicated outcomes were determined.

**Results:**

After a mean follow‐up of 394 days, in 94.4% of the included patients kidney function changed ranging from −30 mL/min to 30 mL/min. The incidence of stroke/SE and major bleeding was similar between patients with deteriorated (reduction in eGFR from baseline over follow‐up) and preserved kidney function change (increase or no change in eGFR from baseline over follow‐up) [stroke/SE: incidence rate (IR): 1.33%/year vs 1.80%/year; hazard ratio (HR) 0.74, 95% confidence interval (CI) 0.41‐1.32, *P* = .30; major bleeding: IR 1.63%/year vs 1.49%/year, HR 1.10, 95% CI 0.61‐1.97, *P* = .76]. On Cox regression analysis, patients with deteriorated kidney function were at higher risk for all‐cause death, compared to patients with preserved kidney function (HR: 1.64, 95% CI: 1.02‐2.63, *P* = .04).

**Conclusion:**

In the BOREALIS trial, the risk of adjudicated stroke/SE, major bleedings, and all‐cause death was not related to mild‐moderate follow‐up changes in kidney function (±30 mL/min). The risk of all‐cause death was significantly increased in AF patients with abruptly deteriorating kidney function.

## Graphical abstract

Uncertainty exists among specialists on the best use of anticoagulation in AF patients with concomitant chronic kidney disease, particularly since kidney function may deteriorate in those taking anticoagulation, especially warfarin. Our analysis showed that all‐cause mortality was significantly higher in anticoagulated AF patients whose kidney function abruptly decreased over the study treatment period. The risk of thromboembolism and major bleedings was not related to mild‐moderate changes in kidney function (±30 mL/min).

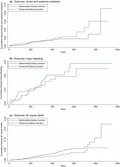

## 1 INTRODUCTION

Atrial fibrillation (AF) and chronic kidney disease (CKD) often complicate each other^1^ and there is evidence that CKD markedly increases the risk of stroke (even with the use of oral anticoagulation),^2^ as well as bleeding and mortality in AF patients.^3^, ^4^ Uncertainty exists among specialists on the best use of anticoagulation in AF patients with concomitant CKD.^5^ As there are few studies on the relationship between deterioration of kidney function and the adjudicated outcomes of anticoagulation in patients with AF.

We performed a secondary analysis of evaluation of Weekly Idrabiotaparinux Sodium Versus Oral Adjusted‐dose Warfarin to Prevent Stroke and Systemic Thromboembolic Events in Patients with Atrial Fibrillation (BOREALIS) trial^6^ comparing idrabiotaparinux with vitamin K antagonists for prevention of thromboembolism in patients with AF to address the unclear associations between the relative risk of stroke, major bleeding, and mortality with changes in kidney function in AF.

## 2 MATERIALS AND METHODS

The design and results of the BOREALIS trial have previously been described and details provided in Supplemental Methods.^6^ In brief, the BOREALIS trial was a multicenter randomized trial comparing idrabiotaparinux and dose‐adjusted warfarin for the prevention of thromboembolism in patients with nonvalvular AF.^6^ The trial protocol was approved by the institutional review board and all patients have provided written informed consent. The trial was stopped early, and showed a comparable effect in stroke and SE prevention of once‐weekly s.c. idrabiotaparinux to that of warfarin, but with inconclusive noninferiority because the upper 95% confidence limit of the primary efficacy outcome (stroke/SE,1.66) was above the prespecified margin for noninferiority (1.38).

To be included in the present analysis: (a) patients should have baseline creatinine measurements; and (b) last creatinine measured within 90 days before the end of the study treatment or after the end of the study treatment. Consequently, patients were divided into two groups according to their kidney function changes, from baseline to the end of the follow‐up, calculated using chronic kidney disease epidemiology collaboration (CKD‐EPI) equation. Changes in kidney function were categorized as “preserved” (ie, increase or no change in eGFR from baseline over follow‐up) or “deteriorated” (reduction in eGFR from baseline over follow‐up). The “deteriorated” group included all individuals who had any level of deterioration in kidney function. Those with kidney function deterioration ≥40 mL/min were defined as abrupt deterioration.

### 2.1 Estimation of kidney function

Three widely used formulae were used to estimate the kidney functions in each patient, as follows:

1. Chronic Kidney Disease Epidemiology Collaboration (CKD‐EPI)^7^ = 141 × min (Scr/κ, 1)^α^ × max(Scr/κ,1)^−1.209^ × 0.993^Age^ × 1.018 (if female) × 1.159 (if African American), where *κ* is 0.7 for women and 0.9 for men, *α* is −0.329 for women and −0.411 for men, min indicates the minimum of Scr/*κ* or 1, max indicates the maximum of Scr/*κ* or 1, and Scr indicates serum creatinine levels, expressed in milligrams per deciliters;
2. The modification of diet in renal disease (MDRD)^8^ GFR (mL/min/1.73 m^2^) = 175 × (Scr)^−1.154^ × (Age)^−0.203^ × (0.742 if female) × (1.212 if African American); and
3. The Cockcroft ‐Gault Equation^9^ = (140 − age) × (Weight in kilograms) × (0.85 if female)/(72 × Creatinine in milligrams per deciliter).

### 2.2 Study endpoints

In this analysis we included all study outcomes, collected from the baseline to the end of the study. The primary analysis of the BOREALIS trial reported only outcomes collected during the treatment period.^6^ The primary efficacy outcome of this analysis was the composite of stroke and systemic embolism (SE). The principal safety outcome of the present analysis was major bleeding, the definition of which was based on the criteria published.^6^, ^10^ All‐cause death was also studied in this study.^6^, ^10^ Suspected outcome events were adjudicated by an independent central adjudication committee who were blinded to treatment assignment.

### 2.3 Statistical analysis

Baseline characteristics were reported as numbers (percentages of actual numbers) or mean ± standard deviation (SD). Continuous variables between two kidney function change groups were compared using independent *t* test. Categorical variable comparisons between kidney function changes were conducted using Chi‐squared test or Fisher's exact test. Univariate Cox regression analysis was used to estimate the hazard ratio of the outcomes of kidney function changes in different settings of the whole cohort, cohort complicated with diabetes mellitus (DM), hypertension (HPT), and heart failure (HF). Event rates (number per 100 patient‐years) were also reported. Multivariate Cox regression analysis adjusted for age, sex, history of hypertension, diabetes, congestive heart failure and/or left ventricular impairment, prior stroke/SE, concomitant aspirin use, concomitant clopidogrel or ticagrelor use, and separately adjusted for kidney function changes (calculated with CKD‐EPI eGFR, MDRD eGFR and CRCL, separately) or for baseline kidney function (calculated with CKD‐EPI eGFR). Restricted cubic spline lines were used to explore the relationship of continuous kidney function changes with the outcomes of stroke/SE, major bleeding, and all‐cause death. Data were considered as missing data if information was unavailable. Kaplan‐Meier curves of outcomes in relation to different kidney function changes were assessed. Analyses were performed using SPSS (version 23.0) and STATA 13 (STATA Inc) softwares. Two‐tailed *P* value < .05 was considered as statistically significant.

## 3 RESULTS

After a follow‐up of 394 [208‐531] days, 2765 participating AF patients in the BOREALIS trial had both measured creatinine variables at baseline and the end of study treatment. Baseline characteristics were compared between the deteriorated and preserved kidney function change (calculated using CKD‐EPI eGFR) groups in AF patients randomized to the two anticoagulant treatment arms (Table 1). Those with deteriorated kidney function change had a tendency to have a higher mean HAS‐BLED score and lower kidney function, but other baseline characteristics were not significantly different. Abrupt kidney function reduction of ≥40 mL/min occurred in 29 patients, which was 1.8% of the deteriorated group.

**Table 1:** Baseline Characteristics

	Deteriorated kidney function change	Preserved kidney function change	*P* value
Age			.55
N	1580	1185	
Mean ± SD	69.36 ± 9.68	69.14 ± 9.77	
Sex, N (%)			.55
Female	596 (37.7)	461 (38.9)	
Male	984 (62.3)	724 (61.1)	
BMI			.67
N	1579	1180	
Mean ± SD	29.98 ± 6.20	29.88 ± 5.97	
AF type, N (%)			.3
Paroxysmal AF	446 (28.3)	304 (25.9)	
Persistent AF	289 (18.3)	234 (19.9)	
Permanent AF	843 (53.4)	638 (54.3)	
Previous HPT, N (%)			.65
Yes	1480 (93.7)	1104 (93.2)	
No	100 (6.3)	81 (6.8)	
LV dysfunction, N (%)			.64
Yes	899 (56.9)	686 (57.9)	
No	680 (43.1)	499 (42.1)	
DM, N (%)			.83
Yes	546 (34.6)	404 (34.1)	
No	1034 (65.4)	781 (65.9)	
CAD, N (%)			.79
Yes	574 (38.9)	421 (38.3)	
No	900 (61.1)	677 (61.7)	
Previous stroke, TIA or non‐CNS systemic embolism, N (%)			.24
Yes	501(31.7)	350 (29.5)	
No	1079 (68.3)	835 (70.5)	
Concomitant‐aspirin, N (%)			.14
Yes	475 (30.1)	325 (27.4)	
No	1105 (69.9)	860 (72.6)	
Anticoagulation type, N (%)			.88
VKA	793 (50.2)	591 (49.9)	
Idraparinux	787 (49.8)	594 (50.1)	
Hb			1.00
N	1564	1165	
Mean ± SD	140.54 ± 16.08	142.86 ± 16.05	
Creatinine			<.001
N	1580	1185	
Mean ± SD	89.44 ± 23.29	102.25 ± 25.89	
CKD‐EPI eGFR			<.001
N	1580	1185	
Mean ± SD	71.25 ± 18.01	61.59 ± 16.93	
≤30	10 (0.6)	13 (1.1)	
30‐60	432 (27.4)	591 (49.9)	
≥60	1138 (72.0)	581 (49.0)	
CHA_2_DS_2_‐VASc score, N (%)			.42
0‐1	1 (0.06)	3 (0.3)	
2	156 (9.9)	110 (9.3)	
3	362 (22.9)	271 (22.9)	
4	434 (27.5)	353 (29.8)	
5	327 (20.7)	255 (21.5)	
≥6	299 (18.9)	193 (16.3)	
HAS‐BLED score, N (%)			.04
0	154 (9.8)	129 (10.9)	
1	504 (31.9)	427 (36.1)	
2	566 (35.8)	417 (35.2)	
3	274 (17.4)	164 (13.9)	
≥4	81 (5.1)	47 (4.0)	

Categorical variables were presented as number (%); Continuous variables were presented as mean ± standard deviation (SD).

CHA2DS2‐VASc, congestive heart failure, 1 point; hypertension, 1 point; age ≥ 75 years, 2 points; diabetes mellitus,1 point; stroke, 2 points; vascular disease, 1 point; age from 65 to 74 years, 1 point; and female sex, 1 point. HAS‐BLED, uncontrolled hypertension (>160 mmHg systolic), 1 point; abnormal renal function, 1 point; or abnormal liver function, 1 point; Prior history of stroke, 1 point; Prior major bleeding or predisposition to bleeding, 1 point; labile INR, 1 point; age > 65 years, 1 point; prior Alcohol or Drug Usage History (≥ 8 drinks/week), 1 point; Medication Usage Predisposing to Bleeding: (Antiplatelet agents, NSAIDs), 1 point.

AF, atrial fibrillation; BMI, body mass index; CAD, coronary artery disease; CKD‐EPI eGFR, Chronic Kidney Disease Epidemiology Collaboration estimate glomerular filtration rate; CNS, central nervous system; DM, diabetes mellitus; HPT, hypertension; LV, left ventricular; TIA, transient ischemic attack; VKA, vitamin‐K antagonist.

### 3.1 Kidney function changes

The distribution of kidney function changes, calculated using three calculation methods and then categorized by 10 unit increments is shown in Figure S1. Of note, 94.4% (calculated using CKD‐EPI eGFR), 92.7% (MDRD eGFR), and 91.6% (CRCL) of kidney function changes ranged from −30 to 30 mL/min, while 77.8% of kidney function changes (CKD‐EPI eGFR), 73.6% (MDRD eGFR), and 73.8% (CRCL) ranged from −15 to 15 mL/min.

### 3.2 All‐cause deaths

All‐cause deaths included 9 strokes, 1 non‐CNS systemic embolism, 4 myocardial infarctions, 5 intracerebral hemorrhages, 3 subdural hematomas, 3 gastro‐intestinal bleedings, 28 other cardiovascular deaths, and 25 other causes (cancer or sepsis). Figure S2 lists the distribution of all‐cause deaths according to CKD‐EPI estimate glomerular filtration rate (eGFR) changes categorized by 10 mL/min increments. Stroke (other than haemorrhage stroke [HS])/SE mainly occurred in AF patients with CKD‐eGFR changes from −20 to 30 mL/min, while other cardiovascular deaths (excluding myocardial infarction [MI]) and other causes of death (mainly sepsis and cancer) mainly from −80 to −20 mL/min.

### 3.3 Thromboembolic outcomes according to kidney function changes

The incidence of stroke/SE events was similar between deteriorated (incidence rate (IR) 1.33) and preserved kidney function change (IR 1.80) (hazard ratio (HR): 0.74, 95% confidence interval (CI) 0.41 − 1.32, *P* = .30) (Figure S3). There were consistent effects with nonsignificant differences between deteriorated and preserved kidney function changes in different settings of DM, HPT, and HF. Adjusted Cox regression analysis found no effect taking kidney function changes as continuous variables, whether calculated using CKD‐EPI, MDRD, or Cockcroft‐Gault equations (Table 2). There was no statistically significant association between the baseline kidney function, calculated by CKD‐EPI equation, and thromboembolic events (HR: 1.02, 95% CI: 1.00‐1.04, *P* = .06), in the separate adjusted Cox analysis.

**Table 2:** Multivariate Cox regression analysis

	CKD‐EPI eGFR change		MDRD eGFR change		CRCL change	
	HR (95% CI)	*P* value	HR (95% CI)	*P* value	HR (95% CI)	*P* value
Stroke/SE	1.00 (0.97‐1.03)	.95	1.01 (0.99‐1.03)	.17	1.00 (0.98‐1.03)	.70
Major bleeding	0.99 (0.96‐1.02)	.49	0.99 (0.97‐1.01)	.50	1.00 (0.97‐1.02)	.85
All‐cause Death	0.96 (0.94‐0.98)	<.001	0.98 (0.97‐0.99)	<.001	0.98 (0.96‐0.99)	.005

CKD‐EPI eGFR, Chronic Kidney Disease Epidemiology Collaboration estimate glomerular filtration rate; CRCL, the Cockcroft‐Gault equation; MDRD eGFR, The modification of diet in renal disease estimate glomerular filtration rate; SE, systemic embolism.

### 3.4 Bleeding outcomes according to kidney function changes

The incidence of major bleeding events was similar between preserved (IR 1.63%/year) and deteriorated (IR 1.49%/year) kidney function changes in the whole group (HR: 1.10, 95% CI: 0.61‐1.97, *P* = .76).

Consistency in bleeding risk was also seen in subgroups including DM, HPT, and HF. There were no significant associations between the changes in kidney function, calculated using the three estimation methods, and the risk of major bleeding (Table 2). A separate Cox model, adjusted for the baseline kidney function, did not show an association with the risk of major bleeding (HR: 0.99, 95% CI: 0.97‐1.01, *P* = .26).

Patients with a deteriorated change in kidney function were at higher risk for all‐cause death (HR: 1.64, 95% CI: 1.02‐2.63, *P* = .04), particularly in those complicated with hypertension (HR: 1.79, 95% CI: 1.07‐2.98, *P* = .03) and heart failure (HR: 1.91, 95% CI: 1.03‐3.56, *P* = .04). The annualized incidence of all‐cause death in those with a deteriorated kidney function change was 3.19% per year compared to those with preserved kidney function change, being 1.94% per year (Figure S3).

**Figure 1:**
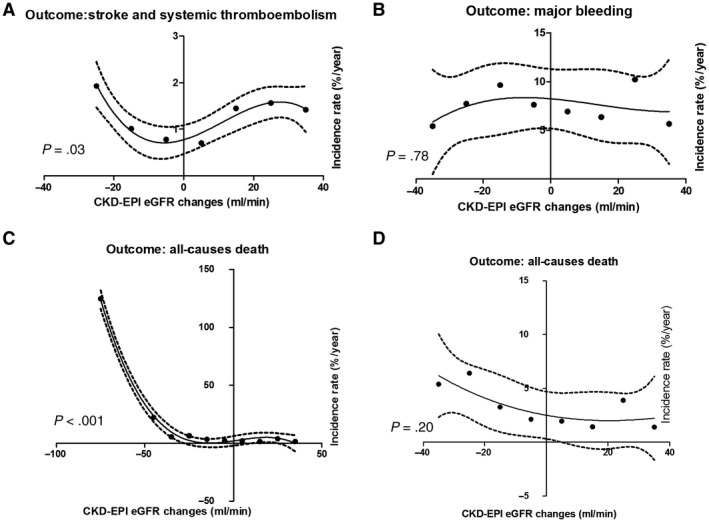
Restricted cubic spline for (A) stroke and systemic embolism, *R* ^2^ = 0.94; (B) major bleeding, *R* ^2^= 0.22; (C) all‐cause death, (with kidney function changes ranging from −80 to 40 mL/min) *R* ^2^ = 0.995 (D) all‐cause death (with kidney function changes ranging from −40 to 40 mL/min), *R* ^2^ = 0.65 with analysis of estimated kidney function changes from baseline to the end of treatment categorized by 10 units with CKD‐EPI

Cox regression analysis showed negative independent associations between kidney function changes, assessed by all three estimation methods, and all‐cause death (Table 2). Baseline kidney function, calculated by CKD‐EPI eGFR formula, did not show independent association with all‐cause death (HR: 1.00, 95% CI: 0.98‐1.01, *P* = .82). As demonstrated in Figure 1, the incidence rate of all‐cause death decreased with improved kidney function during the study treatment period (*P* < .001). If we restricted the analysis to those patients with mild‐moderate changes in kidney function, that is, after excluding those with abrupt changes in kidney function (from −80 to −40), no significant difference was found between kidney function changes ranging from −40 to 40 mL/min and all‐cause death (*P* = .20). In 1041 patients, where kidney function deteriorated to CKD (eGFR < 60 mL/min) level by the end of follow‐up period, the risk of all‐cause death was increased (HR: 1.65, 95% CI: 1.06‐2.57, *P* = .03) with no significant effect on the risk of major bleeding (HR: 1.65, 95% CI: 0.93‐2.95, *P* = .09) and stroke/SE (HR: 0.75, 95% CI: 0.40‐1.40, *P* = .36).

**Figure 2:**
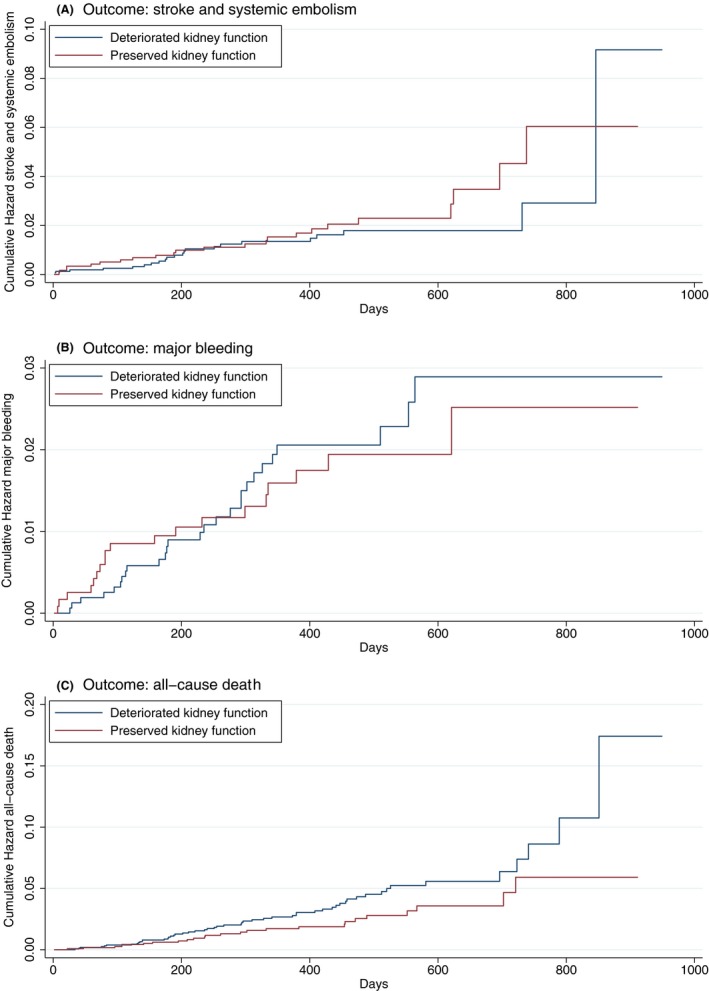
Kaplan‐Meier analysis for risk of stroke and systemic embolism, major bleeding, and all‐cause death. (A) stroke/systemic embolism, (log‐rank: *P* = .30); (B) major bleeding, (log‐rank: *P* = .76); (C) all‐cause death (log‐rank: *P* = .04)

Kaplan‐Meier analysis shows that patients with preserved kidney function changes had a similar risk of stroke/ SE, (log‐rank: *P* = .30), major bleeding, (log‐rank: *P* = .76), but lower risk of all‐cause death (log‐rank: *P* = .04) compared to those with a deteriorated kidney function change (Figure 2).

## 4 DISCUSSION

In the BOREALIS trial population, the risk of all‐cause mortality was significantly higher in anticoagulated AF patients whose kidney function decreased abruptly over the study treatment period. Stroke/SE, major bleedings, and all‐cause death were not related to mild‐moderate changes in kidney function (±30 mL/min).

There remains a dilemma balancing stroke/SE and major bleeding when oral anticoagulation is used in patients with concurrent AF and CKD.^11^ Patients with AF are also inclined to develop kidney dysfunction.^12^, ^13^ However, no significant difference in the risk of stroke/SE and major bleeding was seen in the present study between deteriorated and preserved kidney function during the anticoagulation treatment period, with similar kidney function change between VKA and idrabiotaparinux. One reason was perhaps the narrow range of kidney function changes from baseline to the end of study treatment, as more than 70% of included patients had kidney function changes (calculated using three formulae) within 15 mL/min (from −15 to 15 mL/min) and more than 90% within 30 mL/min (from −30 to 30 mL/min). Previous meta‐analyses^2^, ^3^ showed no significant increase in risk of major bleeding and stroke/SE if kidney function deteriorated within 30% of the baseline kidney function,^14^, ^15^ the conclusion of which was further confirmed by our study because of similar kidney function change scope.

In previous studies of AF patients using warfarin for anticoagulation, kidney dysfunction was a risk factor for death.^16^ However, no significant differences between baseline kidney function and mortality were found in the study of Marcos et al.^17^ In our analysis, we assessed kidney function using three different formulae and found that an *abrupt* deteriorated change in kidney function over time was associated with an increased risk of all‐cause mortality. If study was restricted to those patients with mild‐moderate changes in kidney function (ranging from −40 to +40 mL/min), no significant association was found between kidney function changes and all‐cause mortality. Also, other cardiovascular deaths (excluding myocardial infarction [MI]) and other causes of death (mainly sepsis and cancer) constituted most of “all‐cause deaths” in AF patients with CKD‐eGFR changes from −80 to −40 mL/min (Figure S2).

Subgroup analyses found similar risks of stroke/SE and major bleeding in patients with deteriorated and preserved kidney function with DM, HPT, and HF. Preserved kidney function was associated with a lower risk of all‐cause death compared with deteriorated kidney function in patient subgroups with HF and hypertension, but not in those with DM.

### 4.1 Limitations

The strength of this analysis is the adjudicated clinical outcomes from the posthoc analysis of this trial cohort, but there are several limitations to be mentioned for this study. First, these posthoc analyses from the BOREALIS trial should be interpreted as hypothesis generating only, especially given the limited statistical power for some smaller subgroups. Second, most of the changes in kidney function ranged from −30 to 30 mL/min, and the majority of AF patients enrolled had relatively stable kidney function. Therefore, the results should be cautiously extrapolated to those patients with sharp changes in kidney (dys)function. Third, the underlying pathogenic mechanism(s) were not explored. Finally, we cannot account for the multiple drug therapy changes (and dose alterations) over the follow‐up period.

## 5 CONCLUSIONS

In this trial cohort of anticoagulated AF patients, the risk of all‐cause death was significantly increased in AF patients showing abruptly deteriorating kidney function. There were no significant effects on the risk of stroke/SE, major bleeding, and all‐cause death were seen when kidney function changed from −30 to 30 mL/min.

## CONFLICT OF INTEREST

GYHL has served as a consultant for Bayer/Janssen, BMS/Pfizer, Biotronik, Medtronic, Boehringer Ingelheim, Microlife, and Daiichi‐Sankyo. Speaker for Bayer, BMS/Pfizer, Medtronic, Boehringer Ingelheim, Microlife, Roche and Daiichi‐Sankyo. Other authors: None declared.

## AUTHOR CONTRIBUTIONS

YB takes responsibility for the integrity of the data and the accuracy of the data analysis, and writing of the manuscript. AS and GYHL had full access to all of the data in the study, contributed substantially to the study design, data analysis and interpretation, and the writing of the manuscript.
