# Supplementary material for: Clinical outcomes associated with kidney function changes in anticoagulated atrial fibrillation patients: An ancillary analysis from the BOREALIS trial
Source: J Arrhythm. 2020 Feb 6;36(2):282–8. doi: 10.1002/joa3.12306 (PMC7132193; doi:10.1002/joa3.12306)
Supplement: Supplementary file 1 [file JOA3-36-282-s001.doc]

**ONLINE SUPPLEMENTARY**

**Clinical outcomes associated with kidney function changes in anticoagulated atrial fibrillation patients: An ancillary analysis from the BOREALIS trial**

Ying Bai, PhD ; Alena Shantsila, PhD; Gregory Y.H. Lip, MD, FRCP

| Supplementary Methods | Page 2 |
| --- | --- |
| Supplementary Figure | Page 4 |

**Supplementary Methods**

**The BOREALIS trial** was a multi-center randomized trial comparing idrabiotaparinux and dose-adjusted warfarin for the prevention of thromboembolism in patients with non-valvular AF.

Eligibility criteria were permanent, persistent or paroxysmal, non-valvular, electro-cardiogram(ECG)-documented AF with an indication for long-term VKA therapy based on the presence of previous ischemic stroke, transient ischemic attack (TIA), or SE and/or at least two of the following risk factors: hypertension requiring drug treatment; moderately or severely impaired left ventricular function and/or heart failure; age ≥ 75 years; or diabetes mellitus.

Patients were excluded if they were younger than the legal age to provide informed consent in their country or unable or unwilling to provide informed consent.

Patients were ineligible if they met one or more of the following criteria: baseline creatinine clearance < 30 mL/min (determined locally); indication for VKA other than AF (including prosthetic heart valves or venous thromboembolism); stroke or TIA within 5 days; transient AF caused by a reversible disorder; planned major surgery or cardioversion within 30 days; INR > 3 at baseline; history of intracranial, intraocular, spinal, overt gastrointestinal, retroperitoneal, or traumatic intra-articular bleeding or life-threatening bleeding; active bleeding or high risk of bleeding; uncontrolled hypertension (systolic blood pressure > 180 mm Hg) and/or diastolic blood pressure > 110 mm Hg; any other contraindication listed in the local labeling of warfarin; previous exposure to idraparinux; or known allergy to idrabiotaparinux, avidin, or egg proteins. Women who were pregnant, breastfeeding, or premenopausal and not using effective contraceptive measures were also excluded.

**
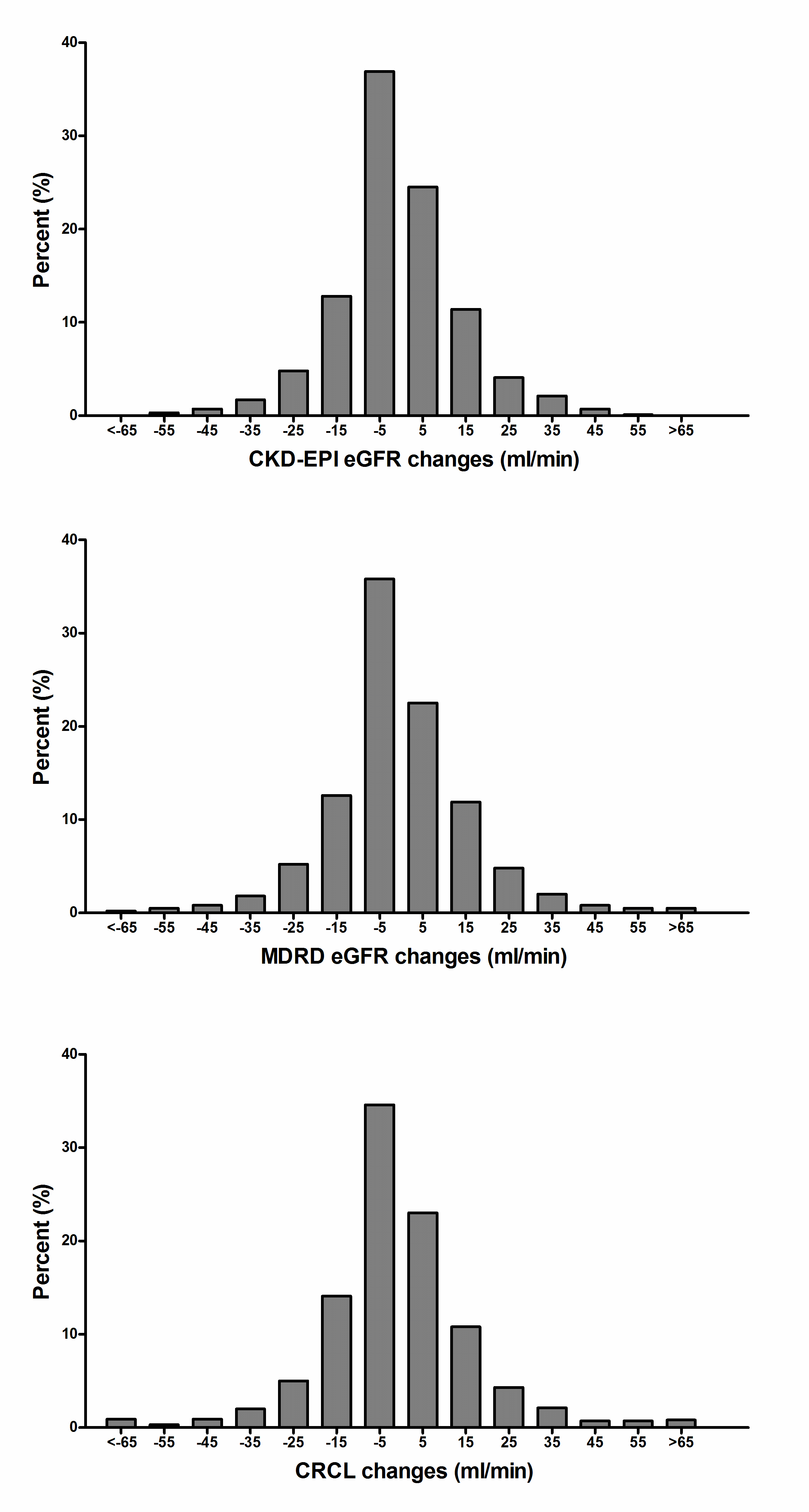
**

**Supplementary Figure 1.** Distribution of kidney function changes from baseline to the end of study treatment calculated using CKD-EPI eGFR, MDRD eGFR and CRCL.

CKD-EPI eGFR, Chronic Kidney Disease Epidemiology Collabotation estimate glomerular filtration rate; MDRD eGFR, The modification of diet in renal disease estimate glomerular filtration rate; CRCL, the Cockcroft –Gault equation.


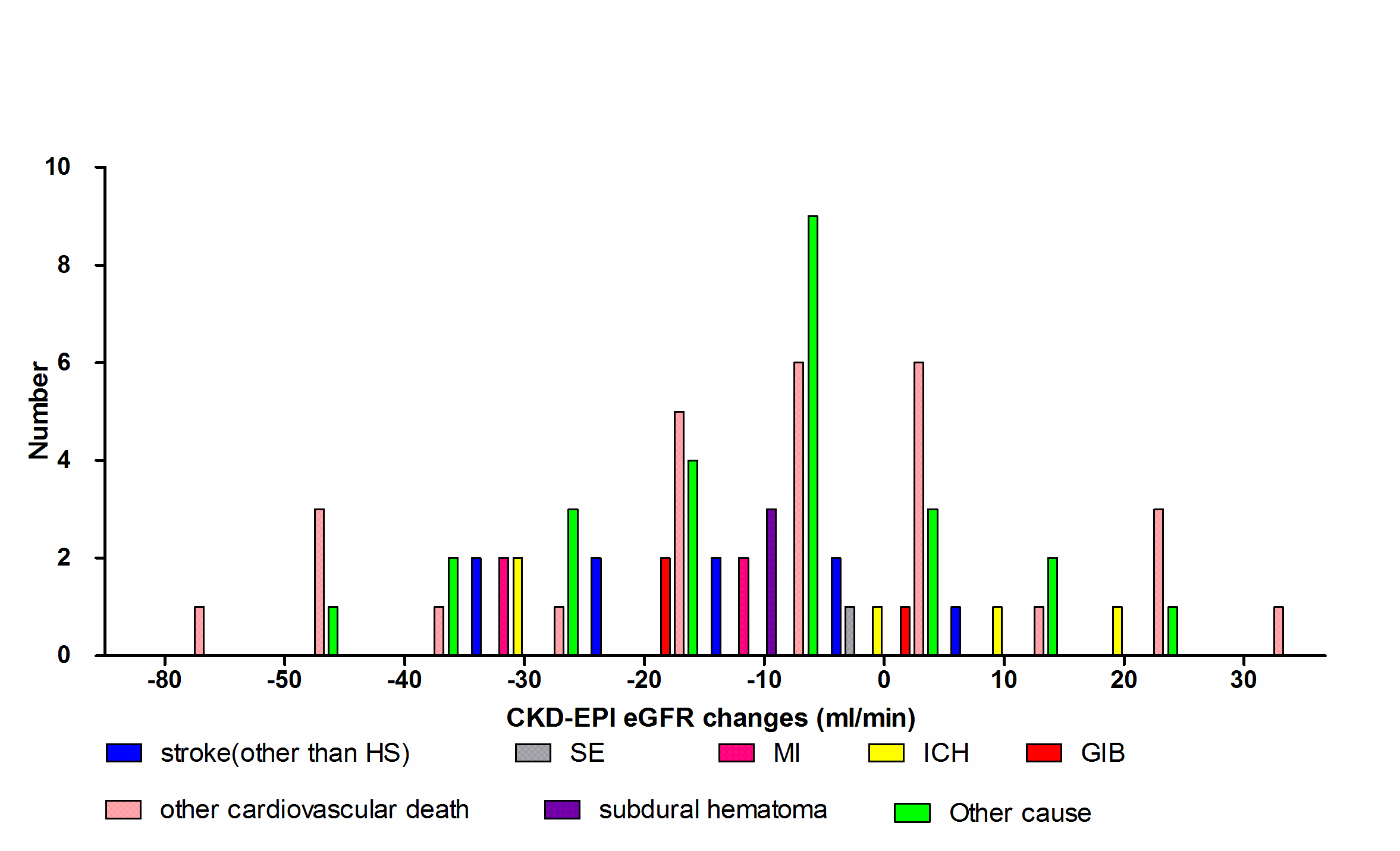


**Supplementary Figure 2.** Distribution of causes of death based on CKD-EPI eGFR change categorized by 10 units (ml/min).

**Supplementary Figure 3.** Forrest plot for effect of deteriorated vs. preserved kidney function for outcomes of stroke and systemic thromboembolism, major bleeding and all-cause death in different settings of the whole group, with DM, HPT and HF. P values are based on the comparison of deteriorated vs. preserved kidney function.

SE, systemic embolism; DM, diabetes mellitus; HPT, hypertension; HF, moderately or severely impaired left ventricular function and /or congestive heart failure; HR, hazard ratio.
